# Spontaneous rupture of non-parasitic or non-neoplastic multiple and giant liver cysts: report of a case

**DOI:** 10.1186/s40792-015-0044-2

**Published:** 2015-05-29

**Authors:** Takehiro Maki, Makoto Omi, Hiroyuki Kaneko, Kenjiro Misu, Hitoshi Inomata, Kazuyoshi Nihei

**Affiliations:** Department of Surgery, Kushiro Red Cross Hospital, 21-14, Shineichyo, Kushiro, Hokkaido 085-8512 Japan

**Keywords:** Rupture, Liver cyst, Polycystic liver, Isolated polycystic liver disease

## Abstract

Simple liver cysts occasionally cause pressure symptoms of the abdomen. We herein report an extremely rare case of spontaneous rupture of simple liver cysts. A 65-year-old woman suffered abdominal fullness and dyspnea. Laboratory examinations revealed general inflammation and mild hepatorenal dysfunction. Computed tomography revealed giant polycystic liver and ascites. Echinococcus antibody was not detected. Abdominal paracentesis provided dark brown transparent ascites in which any parasites or tumor cells were not observed. We diagnosed spontaneous rupture of isolated polycystic liver disease (PCLD) and continuously drained the ascites. After the symptoms and laboratory data were improved, resection of liver cysts and left lateral segmentectomy were performed. Histopathologically, simple columnar epithelia inside of cyst walls were observed. The patient remains well without recurrence of the symptoms 10 months after the surgery. We reviewed characteristics of PCLD and considered appropriate treatment for spontaneous rupture of simple liver cysts based on the previous case reports including the present case.

## Background

Simple liver cysts occasionally cause pressure symptoms of the abdomen and require decompression. We report an extremely rare case of spontaneous rupture of multiple and giant liver cysts, followed by a review of the literature.

## Case presentation

**Fig. 1:**
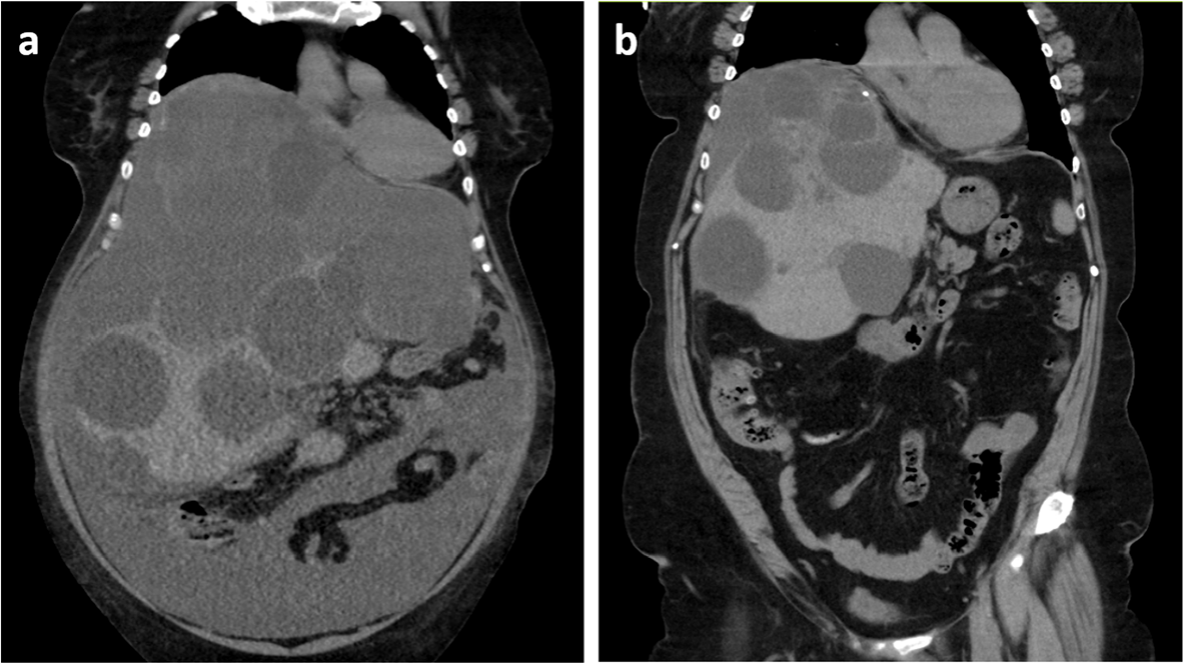
Computed tomography images (frontal section). **a** At the first visit of the patient, giant polycystic liver, ascites, and elevated right diaphragm were observed. **b** Ten months after the surgery, the volume of liver cysts was greatly reduced and ascites were not observed

A 65-year-old woman suffered general fatigue, abdominal fullness, and dyspnea for a month and visited our hospital. The dyspnea had prevented her from sleeping for several days. She had hypertension and underwent laparoscopic cholecystectomy for cholecystitis 20 years earlier. She denied history of trauma. She was 146 cm tall and weighted 97 kg (body mass index, 45.5). Her blood pressure, pulse, and oxygen saturation were 96/59 mmHg, 85 beats/min, and 90 %, respectively. Her body temperature was 37.5 °C. Her abdomen was highly distended, and she could not lie supine because she felt harder to breathe. Abdominal tenderness, muscular defense, or Blumberg’s symptom were not observed. Laboratory examinations revealed general inflammation (leukocyte count, 15,700/μl; C-reactive protein level, 16.2 mg/dl), mild liver dysfunction (total bilirubin, 2.97 mg/dl; direct bilirubin, 0.81 mg/dl), and mild renal dysfunction (creatinine, 1.40 mg/dl). Anemia was not indicated (hemoglobin level, 13.3 g/dl). Computed tomography revealed giant polycystic liver which occupied the large part of the peritoneal cavity, much ascites in the lower abdomen, and highly elevated right diaphragm (Fig. 1a). Fractional liver parenchyma was detectable in the right lobe. Only two small cysts were observed in kidneys. Serological tests for Echinococcus antibody were negative. Abdominal paracentesis provided dark brown transparent ascites that showed no evidence of parasites or neoplasms. Any kinds of bacteria were not detected by culture of the ascites.

We diagnosed isolated polycystic liver disease (PCLD) and spontaneous rupture of the cysts. The cause of leukocytosis and high serum CRP values was not apparent, but we denied infectious peritonitis on the ground of physical findings and appearance of the ascites. We continuously drained the ascites whose volume was totally 16,000 ml in 25 days. Her dyspnea, general inflammation, and hepatorenal dysfunction were improved (oxygen saturation, 97 %; leukocyte count, 5920/μl; C-reactive protein level, 6.17 mg/dl; total bilirubin, 0.56 mg/dl; direct bilirubin, 0.17 mg/dl; creatinine, 0.56 mg/dl). Her weight and body mass index downed to 82 kg and 38.5, respectively. Despite those remarkable improvements, abdominal fullness remained after the drainage.

**Fig. 2:**
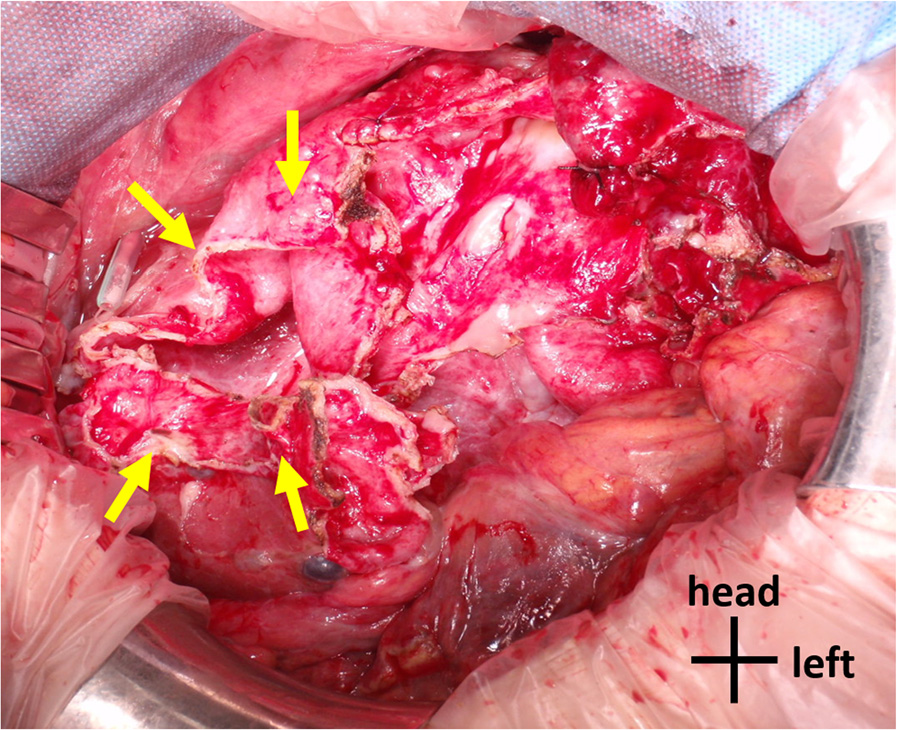
Intraoperative photograph. At laparotomy, the right lobe of the liver is shown. *Arrows* show a surgically unclosed liver cyst. The large part of the liver was replaced to cysts

**Fig. 3:**
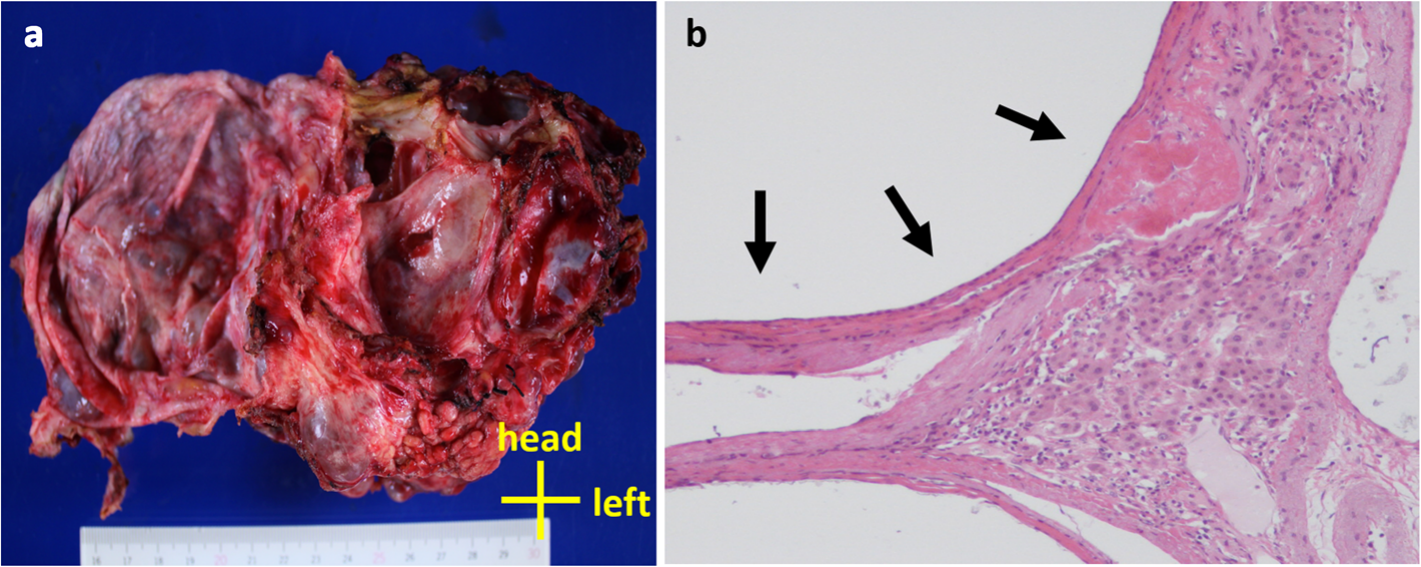
Findings of excised specimens. **a** Photograph of resected left lateral segment. The left lateral segment consisted almost entirely of simple cysts. **b** Microscopic image of excised left lateral segment. The liver cyst wall is consisted of simple columnar epithelia (*arrows*)

Twenty-five days after her visit to our hospital, resection of multiple liver cyst walls and left lateral segmentectomy were performed (Fig. 2). We started the surgery by laparoscopic approach but switched to laparotomy to repair the left hepatic duct which was injured by the laparoscopic manipulation. Kinds of intracystic fluid varied between individual cysts; colorless transparent, dark-brownish transparent, bile-like transparent, or abscess-like muddy fluid were observed. Discharged fluid was totally 7500 ml. On gross examination, excised left lateral segment consisted almost entirely of cysts and resected all cyst walls were thickened without mural nodules (Fig. 3a). Histopathological examination of the specimens revealed simple columnar epithelia inside of cyst walls and fractional liver parenchyma between the cyst walls and showed no evidence of parasites or neoplasms (Fig. 3b). After the surgery, her abdominal fullness was greatly lessened and she has been asymptomatic for 10 months (Fig. 1b).

Simple liver cysts sometimes reach large sizes and cause pressure symptoms resulting from mass effect, vascular compression, and biliary obstruction [1]. In the present case, multiple and giant liver cysts occupied most of the abdomen. Initially, polycystic liver was considered to be associated with autosomal dominant polycystic kidney disease (ADPKD) [2]. In 2003, Tahvanainen et al. indicated that polycystic liver in some patients was genetically distinct from ADPKD; it is now known and described as PCLD [3]. PCLD has an autosomal dominant inheritance pattern as ADPKD does. Genetic mutation in PKD1 or PKD2 was observed in both PCLD and ADPKD, but genetic mutation in PRKCSH or SEC63 was specifically identified only in PCLD [4]. Although genetic examination was not performed, the present case was clinically diagnosed as PCLD according to the diagnostic algorithm devised by Lantinga et al. [5]. The present case provided unexplained inflammation which might reflect that some liver cysts were infectious because several cysts contained abscess-like fluid in the operation. PCLD is a rare condition and has prevalence of less than 0.01 % [6]. Similar to simple liver cysts, cysts in PCLD contain a clear, bile-like fluid, and pathologically consist of an inner lining of cholangiocytes [7]. PCLD can lead to massive hepatomegaly and cause pain or compression of the adjacent gastrointestinal organs, vasculature, and diaphragm [8]. Van Keimpema et al. examined clinical features of PCLD in their retrospective study (*n* = 137) [9]. In summary, they described that symptomatic PCLD patients were mainly females (86 %), most patients had more than 20 liver cysts (88 %), median diameter of the largest cyst was 9 cm, females and mutation carriers were younger at diagnosis and had more severe courses of disease, and PCLD-related mortality was 2 %. Hoevenaren et al. compared the clinical features of patients with PCLD (*n* = 19) with those of patients with ADPKD (*n* = 34) [10]. They concluded that the clinical course of PCLD was relatively benign compared with ADPKD, although PCLD was characterized by larger and greater number of liver cysts. While ADPKD is characterized by an increased risk of developing vascular manifestations such as hypertension, mitral valve prolapse, and intracranial aneurysms, several studies have shown that PCLD patients do not have the risk and targeted screening for those diseases is not advised for PCLD [5, 10]. For PCLD patients with pressure symptoms, surgical reduction of the volume of the liver cysts has been generally performed to diminish the mass effect [11] and somatostatin analogues, lanreotide, and octreotide are expected to relieve the symptoms as optional therapies [8].

Spontaneous rupture of simple liver cysts including polycystic liver is extremely rare, and only 14 cases have been reported including this case [12–24] (Table 1). Median age of onset is 62.5. Male to female ratio of the patients is 5:9. Two patients had received maintenance hemodialysis for chronic renal failure due to ADPKD. Only our case was diagnosed as PCLD. Most of the patients complained of abdominal pain. Locations of ruptured cysts were the right lobe in six patients and the left lobe in eight patients. Median size of the ruptured cyst is 12.5 cm. In the present case, we could not identify a location of the ruptured cyst because our case had many cysts all over the liver and we did not perform an emergency surgery. Four of 14 cases (29 %) had hemorrhage with rupture. In most patients, the liver cyst ruptured into the peritoneal cavity while two cases ruptured into the common hepatic duct and the hepatic subcapsule [22, 24]. As treatments for the rupture of liver cysts, invasive procedures were mainly performed and they varied from percutaneous drainage to hepatectomy. Two of 14 cases (14 %) had recurrence of pressure symptom. In these two cases, marsupialization and percutaneous drainage were performed. In other eight cases, resection of cyst walls or hepatectomy was performed and no recurrence occurred. Basically, palliative reduction is thought to be appropriate for pressure symptoms of simple liver cysts [1], but marsupialization or percutaneous drainage may be insufficient, and more invasive procedures such as cystic wall resection and hepatectomy may be recommended to prevent the recurrence of the symptoms. Only one case died of postoperative serious complications; the case had end-stage renal failure due to ADPKD and life-threatening hemorrhage from a ruptured liver cyst. Spontaneous rupture of simple liver cysts without hemorrhage may lead to favorable prognosis. In the present case, we temporarily carried out percutaneous drainage of the ascites to relieve dyspnea and performed surgical reduction of the liver cysts after general condition was improved and favorable outcome was obtained.

**Table 1:** Reported 14 cases of spontaneous rupture of simple liver cysts

Reference	Age/sex	Comorbidity	Major complaint	Ruptured cyst		Hemorrhage	Treatment	Outcome
				Location	Size (cm)			
1959, Morgenstern [12]	56/F	–	Abdominal pain	Left lobe	35	−	Left lobectomy	No symptoms
1972, Russell [13]	68/M	–	Abdominal pain, vomiting	Left lateral segment	12	−	Left lobectomy	No symptoms
1974, Brunes [14]	54/F	–	Abdominal pain	Left lobe	25	−	Partial removal of the cyst	No symptoms
1988, Ayyash [15]	36/F	–	Abdominal pain, nausea	Segment 5	4	−	Excision of the cyst	No symptoms
1989, Akriviadis [16]	48/F	–	Abdominal pain	Left lateral segment	Not described	−	Observation	No symptoms
1998, Chung [17]	76/F	ADPKD	Abdominal pain	Right lobe	16.7	+	Marsupialization	3 months, wide excision of the cyst for recurrence
1999, Yamaguchi [18]	61/M	–	Abdominal pain	Left lobe	13	−	Left trisegmentectomy	No symptoms
2002, Carels [19]	76/M	ADPKD	Abdominal pain	Right lobe	9	+	Hemostasis by placing omentum	1 month, dead
2002, Ishikawa [20]	42/F	–	Abdominal discomfort	Segment 4/5	10	+	Transcatheter arterial embolization, percutaneous transhepatic puncture	3 weeks, cystectomy for recurrence
2007, Salemis [21]	50/M	COPD	Abdominal pain	Left lobe	17	−	Wide excision of the cyst	1 year, no symptoms
2010, Ueda [22]	64/F	–	Abdominal pain	Right lobe	10	−	Percutaneous aspiration, intracystic injection of minocycline hydrochloride	1 year, no symptoms
2010, Miliadis [23]	70/M	–	Abdominal pain	Right lobe	13	−	Unroofing the cyst, omentoplasty	4 days, discharged
2011, Senadhi [24]	91/F	–	Abdominal pain, melena	Segment 4	3	+	Removal of blood clots by endoscopy	1 months, no symptoms
[The present case]	65/F	PCLD	Dyspnea, abdominal distension	Unknown	Unknown	−	Left lateral segmentectomy, resection of the walls of the cysts	10 months, no symptoms

## Conclusions

PCLD is a different disease from ADPKD, tends to have greater and larger cysts and thus cause pressure symptoms more often than ADPKD, and provides better response to surgical reduction than ADPKD. For spontaneous rupture of simple liver cysts, surgical procedures such as cystic wall resection and hepatectomy generally seem to yield favorable outcomes without recurrence of pressure symptoms.

## Consent

Written informed consent was obtained from the patient for publication of this case report and any accompanying images. A copy of the written consent is available for review by the Editor-in-Chief of this journal.

## Abbreviations

PCLD: represents isolated polycystic liver disease
ADPKD: autosomal dominant polycystic kidney disease
COPD: chronic obstructive pulmonary disease
